# Ocular Surface Epithelial Thickness Evaluation in Dry Eye Patients: Clinical Correlations

**DOI:** 10.1155/2016/1628469

**Published:** 2016-01-26

**Authors:** Qingfeng Liang, Hong Liang, Hanruo Liu, Zhiqiang Pan, Christophe Baudouin, Antoine Labbé

**Affiliations:** ^1^Beijing Institute of Ophthalmology, Beijing Tongren Eye Center, Beijing Tongren Hospital, Capital Medical University, Beijing Key Laboratory of Ophthalmology and Visual Sciences, Beijing 100005, China; ^2^INSERM, U968, 75012 Paris, France; ^3^UPMC Université Paris 06, UMR_S 968, Institut de la Vision, 75012 Paris, France; ^4^CNRS, UMR_7210, 75012 Paris, France; ^5^Quinze-Vingts National Ophthalmology Hospital, 75012 Paris, France; ^6^Versailles Saint-Quentin-en-Yvelines University, 78000 Versailles, France

## Abstract

*Purpose.* To evaluate the relationship between corneal and conjunctival epithelium thickness and ocular surface clinical tests in dry eye disease (DED) patients.* Patients and Methods.* Fifty-four patients with DED and 32 control subjects were included. Each patient underwent an ocular surface evaluation using the ocular surface disease index (OSDI), tear film break-up time (TBUT), corneal and conjunctival staining, tear film lipid layer analysis, and Schirmer test. The central corneal (CET), limbal (LET), and bulbar conjunctival epithelium thickness (BET) were acquired using spectral-domain optical coherence tomography (SD-OCT).* Results.* Compared to control subjects, mean BET was significantly thicker and mean LET was significantly lower in the DED group. There was no significant difference in mean CET between the two groups. The mean LET was correlated with OSDI and TBUT. The inferior LET was correlated with OSDI, Schirmer I test, TBUT, Oxford score, and corneal sensitivity. Mean BET was correlated with OSDI and TBUT, but not with Schirmer I test and Oxford score.* Conclusions.* In dry eye patients, a thinner limbal epithelium and a thicker bulbar conjunctival epithelium were observed. These changes were correlated to the severity of dry eye symptoms and tear film alterations.

## 1. Introduction

Dry eye disease (DED) is a multifactorial disease of the tears and ocular surface resulting in tear film instability with potential damage to conjunctival and corneal epithelium [1]. It results from a disturbance of the lacrimal functional unit that includes the tear film, the lacrimal and meibomian glands, the ocular surface epithelium, and the sensory and motor nerves that connect them [2]. Inflammation with inflammatory cell infiltration and cytokine production are also common features of DED, found in the lacrimal glands, the cornea, and the conjunctiva [3]. In association with the mechanical and desiccating stress induced by the lack and/or the poor quality of tears, inflammation further damages ocular surface epithelia. Considering the central role of these tissues in the pathophysiology of DED, several imaging techniques have been developed to evaluate and grade the alterations of ocular surface epithelia* in vivo* [4, 5]. Despite lower resolution than* in vivo* confocal microscopy (IVCM), SD-OCT has numerous advantages over other imaging techniques, such as slit-lamp or ultrasound biomicroscopy [6]. OCT is a noninvasive imaging method that allows high-resolution analysis and quantification without the need for ocular anesthesia or contact procedures. In a preliminary study, we used SD-OCT to noninvasively evaluate ocular surface epithelial thickness [4]. In DED patients and patients using IOP-lowering eye drops, we observed a decreased limbal-conjunctival epithelial thickness and an increased bulbar conjunctival epithelial thickness. The epithelial thickness measurement of ocular surface tissues with SD-OCT seemed to be an advantageous new parameter during ocular surface evaluation. However, the number of patients in each group was limited and correlations were not evaluated between epithelium thickness changes and ocular surface clinical tests. Thus, the objective of the present study was to compare the results of corneal, limbal, and conjunctival epithelium thicknesses obtained with SD-OCT in normal subjects and non-Sjögren dry eye patients and to evaluate the relationship between these parameters and the results of ocular surface clinical tests.

## 2. Patients and Methods

### 2.1. Subjects

This study was conducted at the Beijing Institute of Ophthalmology with approval of the Medical Ethics Committee of Beijing Tongren Hospital (TREC-2013-KY012). All patients were informed of the aims of the study and their consent was obtained according to the declaration of Helsinki. A total of 54 patients with DED not associated with Sjögren syndrome (36 women and 18 men; mean age: 44.59 ± 10.08 years; range: 24–68 years) were consecutively recruited from the Cornea Unit of Beijing Tongren Hospital from June 2013 to February 2014 (DED group). The sample size was calculated according to the results of Cui et al.'s study [7] with 80% power level. DED was defined as Schirmer I testing <5 mm and/or tear film break-up time (TBUT) <10 s, accompanied by complaints of ocular irritation in the absence of other ocular (in particular meibomian gland disease) or systemic diseases [1]. Thirty-two age- and gender-matched control subjects (20 women and 12 men; mean age: 43.34 ± 10.81 years; range: 19–67 years) were also recruited (control group). All control subjects had no complaint of ocular surface irritation and no anterior segment abnormality on biomicroscopic examination and ocular surface tests. Exclusion criteria for both groups were as follows: age <18 years, subject unable to complete the questionnaire or understand the procedures, the presence of ocular or systemic disease or the use of topical or systemic medications that may affect the cornea and the ocular surface (except the use of nonpreserved tear substitutes in the DED group), and previous eye surgery or contact lens wear.

### 2.2. Clinical Evaluation

Demographic information and medical history were obtained from the patients' medical records. Each subject underwent quantification of ocular surface symptoms with the Ocular Surface Disease Index (OSDI) questionnaire (range: 0–100). Then, the subjects underwent ocular surface examinations in the following order: tear film break-up time (TBUT), corneal and conjunctival fluorescein staining, tear film lipid layer analysis, Schirmer test without anesthesia, and corneal sensation measured with the Cochet-Bonnet esthesiometer.

TBUT was measured by instilling fluorescein into the inferior cul-de-sac and calculating the average of three consecutive break-up times. Corneal and conjunctival staining was evaluated under a yellow filter using the Oxford scale and after instillation of fluorescein. Tear film lipid layer analysis was performed using interferometry (DR-1, Kowa, Tokyo, Japan) and evaluated semiquantitatively from 1 to 5 (grade 5 being the most severe) [5]. Schirmer I test was performed without anesthesia for 5 min with the patient's eyes closed. Corneal sensation was measured using the contact nylon thread Luneau 12/100 mm Cochet-Bonnet esthesiometer (Luneau, Prunay-Le-Gillon, France) in the central cornea and in the superior, inferior, nasal, and temporal quadrants. Mean corneal sensitivity (MCS) was defined as the mean of the measures obtained in the five different areas.

### 2.3. SD-OCT Examination and Image Analysis

SD-OCT fitted with an anterior segment module (Optovue Corporation, Fremont, CA, USA) was used. This SD-OCT has a scan rate of 26,000 axial scans per second. Its axial and transverse optical resolution were 5 *μ*m and 15 *μ*m, respectively. An add-on lens (CAM-L mode: 6.0–2.0 mm) was used to assess the regional corneal and conjunctival architecture and epithelial thickness. Because SD-OCT examination is a noncontact technique, it was performed before ophthalmological examinations in order to avoid potential epithelium alterations.

**Figure 1:**
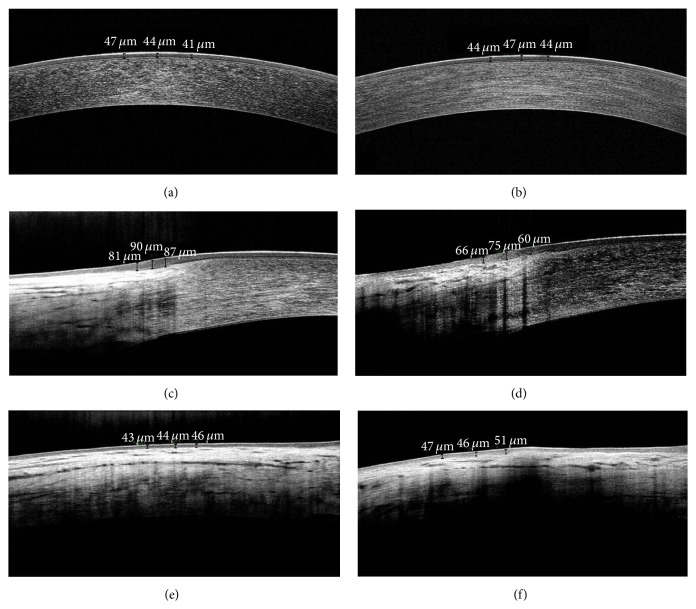
SD-OCT images of ocular surface epithelial thickness measurement in a healthy control subject (a, c, e) and a dry eye patient (b, d, f). Corneal epithelium thickness measurement with software cursors (a, b), limbus and conjunctiva epithelium thickness analysis (c, d), and conjunctiva epithelium thickness measurement (e, f).

The specific imaging capture technique for this study has been previously described [4]. Briefly, patients were asked to fixate on the target light source, and consecutive images were acquired with the patient's forehead and chin stabilized by a headrest. Corneal epithelium thickness (CET) was defined as the epithelium thickness in the 2 mm central zone of the cornea; limbal epithelium thickness (LET) was defined as the limbal-conjunctival epithelium thickness in each quadrant (superior, inferior, temporal, and nasal); bulbar conjunctival epithelial thickness (BET) was defined as the bulbar conjunctival epithelial thickness located between 2 and 3 mm from the limbus of each quadrant (Figure 1).

The cursors were placed perpendicular to the ocular surface epithelium from a point located just beneath the tear film (first hyperreflective layer) to the basal membrane (second hyperreflective layer). For every quadrant, three measurements were taken (if the difference between measurements exceeded 3 *μ*m, the measurement was repeated in order to confirm thickness measurement reproducibility; the measurement needed to be confirmed in less than 5% of cases), and the results were expressed as mean ± SD. The measurements were taken by one researcher (HL) who was masked to patient demographic data and the results of ophthalmologic examinations. To evaluate interobserver variability, a second examiner (QL), who was masked to the results of the first SD-OCT analyses, assessed CET, LET, and BET from the same images of 20 randomized patients.

### 2.4. Statistical Analysis

Statistical analyses were performed with SPSS for Windows version 16.0 (SPSS Inc., Chicago, IL, USA). For each patient, one eye was randomly chosen for statistical analysis. The mean ± SD values of each epithelial thickness variable were calculated for both the DED and control groups. To compare ocular surface parameters and epithelial thickness, measured in normal and DED eyes, two-tailed Student's *t*-tests were performed. The Pearson correlation coefficient was used in DED patients to investigate the correlation between the quantitative measurements of epithelial thicknesses and the results of other ocular surface evaluations. *P* values less than 0.05 were considered statistically significant.

## 3. Results

There was no difference in terms of gender (*P* = 0.699) and age (*P* = 0.503) between the DED and control groups. Concerning ocular surface clinical evaluation, DED patients had significantly more symptoms (OSDI) (35.46 ± 14.94 versus 1.52 ± 3.96, *P* < 0.001), lower TBUT (4.67 ± 2.30 s versus 13.31 ± 2.54 s, *P* < 0.001), a lower Schirmer I test score (4.11 ± 6.98 mm versus 12.56 ± 6.99 mm, *P* < 0.001), and a higher Oxford score (1.06 ± 1.73 versus 0.00, *P* = 0.001) as compared to the control group. Mean corneal sensitivity was significantly decreased in the DED group (5.74 ± 0.41 mm versus 5.98 ± 0.07 mm, *P* = 0.002), while tear film lipid layer interferometry was not statistically different between the two groups (2.43 ± 0.71 mm versus 2.28 ± 0.52, *P* = 0.323). Results of clinical data are presented in Table 1.

**Figure 2:**
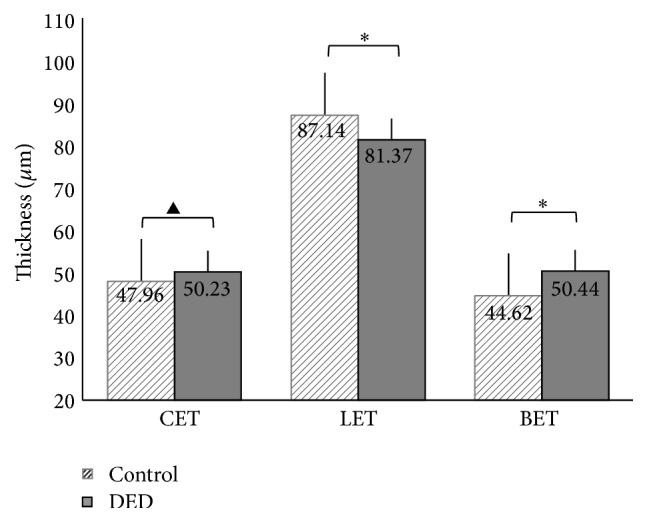
Comparison of CET, LET, and BET data between DED and control group. *∗* represents a significant difference between the indicated groups, *P* ≤ 0.05; ▲ represents no significant difference. ANOVA with Tukey's post hoc test.

Mean CET, LET, and BET were 50.23 ± 4.42 *μ*m, 81.37 ± 6.21 *μ*m, and 50.44 ± 5.25 *μ*m in the DED group and 49.76 ± 3.15 *μ*m, 87.14 ± 9.98 *μ*m, and 44.62 ± 5.04 *μ*m in the control group, respectively. Compared to control subjects, mean BET was significantly thicker (*P* < 0.001) and mean LET was significantly lower (*P* = 0.009) in the DED group. There was no significant difference in CET between the two groups (*P* = 0.103). In addition, dry eyes had a significantly thicker BET in each quadrant region (superior, *P* = 0.005; inferior, *P* = 0.014; temporal, *P* < 0.001; and nasal, *P* = 0.003) than that of normal subjects. In dry eyes, the LET was also significantly thinner in the inferior (*P* = 0.011), temporal (*P* = 0.008), and nasal regions (*P* < 0.001) but not in the superior region (*P* = 0.152) as compared to control subjects (Table 2, Figure 2).

Within the DED group, there were significant correlations between symptoms (OSDI) and Schirmer I test (*r* = −0.312, *P* = 0.003), TBUT (*r* = −0.720, *P* < 0.001), and Oxford score (*r* = 0.340, *P* = 0.001). TBUT was also correlated with Schirmer I test and Oxford score (*r* = 0.436, *P* < 0.001 and *r* = −0.504, *P* < 0.001, resp.). Tear film lipid layer interferometry was correlated with TBUT, Oxford score, and MCS (*r* = −0.236, *P* = 0.029; *r* = −0.389, *P* < 0.001; and *r* = 0.259, *P* = 0.016, resp.) (Table 3).

When evaluating the relationship between dry eye clinical tests and ocular surface epithelium thickness parameters, mean BET was correlated with OSDI (*r* = 0.362, *P* < 0.001) and TBUT (*r* = −0.428, *P* < 0.001) but not with Schirmer I test (*r* = −0.165, *P* = 0.290) and Oxford score (*r* = 0.134, *P* = 0.392). The mean LET was correlated with OSDI and TBUT (*r* = −0.305, *P* = 0.047 and *r* = 0.378, *P* = 0.012, resp.). Interestingly, the inferior quadrant LET was correlated with OSDI (*r* = −0.519, *P* < 0.001), Schirmer I test (*r* = 0.271, *P* = 0.012), TBUT (*r* = 0.638, *P* < 0.001), Oxford score (*r* = −0.256, *P* = 0.017), and MCS (*r* = −0.519, *P* < 0.001) (Table 4).

## 4. Discussion

Several studies have been conducted to measure the thickness and morphology of ocular surface epithelium with OCT, IVCM, or ultrasound, in order to better understand epithelial alterations in DED [7–13]. With Fourier-domain OCT, Cui et al. evaluated for the first time the features of the corneal epithelial thickness map within the central 5 mm zone and the correlation with symptoms in dry eye patients [7]. Yang et al. [14] and our group [4] reported the evaluation with SD-OCT of ocular surface epithelia including the bulbar conjunctival epithelium, the limbal epithelium, and the central corneal epithelium thicknesses in normal subjects and dry eye patients. However, no studies reported the correlations between DED clinical features and ocular surface epithelial thicknesses, including the limbal and the conjunctival epithelium. In accordance with our previous results [4], we observed a thinner limbal epithelium and a thicker conjunctival epithelium in patients with dry eye. Moreover, we observed that the severities of symptoms (OSDI) and tear film alterations (TBUT) were correlated to both LET and BET.

When evaluating the CET of DED patients, some authors observed a decrease, while others found no change or even an increase as compared with the control group [4, 7, 8, 15]. Comparison between studies was made difficult by the different durations and severities of the disease, the varying ages of DED patients, and the different techniques used to measure CET. Fabiani et al. [15] established a mouse model of dry eye and detected that the average CET became significantly thicker in dry eye mice as compared to the controls after 7 days. These results demonstrated that the inflammatory processes and epithelial proliferation had a significant impact on the average CET in the early stage of DED. The studies from Chen et al. [16] and Kanellopoulos and Asimellis [17] indicated that increased epithelial thickness might be used as an objective clinical indicator of dry eye. Conversely, Cui et al. [7] found that the superior corneal epithelium was thinner in DED patients than in normal subjects. Erdélyi et al. [18] and Villani et al. [19] also showed that the CET tends to be thinner in DED patients, which was attributed to the destruction of stem cells at the limbus. In the present study, there was no significant difference in CET between DED patients and the control group, consistent with our previous results [4] and the results from Tuominen et al. [20]. This may be explained by the moderate severity of DED (average OSDI 35.46 and TBUT 4.67 s) and the location of CET evaluation in the central cornea (central 2 mm diameter area), away from the limbus.

Considering the role of limbal epithelial stem cells (LESCs) in corneal epithelium homeostasis, the limbal region is essential in dry eye physiopathology [21]. Several factors might explain the reduced thickness of the limbal epithelium in DED. First, stem cell metabolism can be directly affected in DED patients [22–24]. Infiltration of CD4^+^ T cells at the limbus and the levels of inflammatory cytokines in tears may play an important role in inhibiting corneoscleral stem cell metabolism in dry eye patients. This downregulation of limbal stem cells in DED patients could influence the development of corneal limbal epithelial layers and result in a thinner LET. Increased turnover of corneal epithelial cells might also explain limbal epithelial cell depletion in DED [4]. Limbal microenvironment inflammation could also directly alter limbal stem cells and their functions, resulting in various degrees of stem cell deficiency [25]. The inferior and superior limbal areas are thought to be the largest reservoirs of limbal stem cells as compared to the nasal and temporal quadrants. With confocal microscopy and scanning electron microscopy, Shortt et al. [26] showed more limbal crypts in the superior and inferior limbal regions. Similarly, Thoft et al. found a larger number of stem cells in the superior and inferior limbus than in the medial and lateral areas [27]. Interestingly, in the present study, the inferior LET seemed to be the most sensitive parameter because it was directly correlated to the OSDI, the Schirmer test, TBUT, the Oxford score, and mean corneal sensitivity. The greatest changes observed for the inferior LET could be explained by the prolonged contact between epithelial cells and altered tears within the inferior lacrimal river containing inflammatory factors such as cellular debris or proinflammatory cytokines. Decreased corneal sensitivity correlated to the inferior LET was also observed in DED patients. Corneal nerves are implicated in DED pathophysiology and DED patients exhibit nerves alterations [5]. These nerves changes have been correlated to the severity of the ocular surface lesions and might be in part responsible for corneal sensitivity alterations. As observed with corneal nerves, a thinner inferior LET might represent a marker of DED severity and emphasized the role of LESCs in ocular surface diseases.

The bulbar conjunctiva is an essential tissue of the ocular surface with numerous ocular surface cell populations including inflammatory cells and goblet cells [28, 29]. In this study, the mean BET in dry eye patients was significantly increased as compared to normal eyes. Moreover, the thickness of the conjunctival epithelium layers was directly correlated to symptoms (OSDI) and tear film alterations (TBUT). The infiltration of inflammatory cells and tissue edema observed within the conjunctiva in DED patients might explain, at least in part, the thickening of the conjunctival epithelium [30].

Although the changes in ocular surface epithelium thickness evaluated with SD-OCT are not specific of a particular etiology of DED and may also be observed in other ocular surface diseases, this parameter is providing useful information for the evaluation of ocular surface tissue changes. Given that it is already used for the evaluation of keratoconus, ocular surface epithelial mapping, especially corneal limbus epithelial mapping, might be used in association with other clinical parameters to monitor DED ocular surface changes and the benefit of different treatments in the future.

## Figures and Tables

**Table 1:** Demographic and clinical test results.

Parameters	DED group	Control group	*P*value
Number of patients	54	32	
Gender			
Female	36 (66.7%)	20 (62.5%)	0.699
Male	18 (33.3%)	12 (37.5%)	
Age (years)	44.59 ± 10.08	43.34 ± 10.81	0.503
OSDI	35.46 ± 14.94	1.52 ± 3.96	<0.001
Schirmer I test (mm)	4.11 ± 6.98	12.56 ± 6.99	<0.001
TBUT (seconds)	4.67 ± 2.30	13.31 ± 2.54	<0.001
Oxford scale	1.06 ± 1.73	0.00 ± 0.00	0.001
MCS (mm)	5.74 ± 0.41	5.98 ± 0.07	0.002
TFL	2.43 ± 0.71	2.28 ± 0.52	0.323

DED: dry eye disease; OSDI: Ocular Surface Disease Index; TBUT: tear film break-up time; MCS: mean corneal sensitivity; TFL: tear film lipid layer interferometry.

**Table 2:** Comparison of ocular surface epithelium thickness (*μ*m) between DED and control groups.

Parameters	DED group	Control group	*P* value
Number of patients	54	32	
CET	50.23 ± 4.42	49.76 ± 3.15	0.103
LET	81.37 ± 6.21	87.14 ± 9.98	0.009
LET (N)	80.60 ± 9.27	89.95 ± 15.23	<0.001
LET (T)	80.73 ± 8.91	87.65 ± 11.19	0.008
LET (S)	82.10 ± 8.48	84.64 ± 6.75	0.152
LET (I)	80.12 ± 7.84	86.74 ± 14.45	0.011
BET	50.44 ± 5.25	44.62 ± 5.04	<0.001
BET (N)	50.99 ± 5.26	43.45 ± 4.99	0.003
BET (T)	50.37 ± 6.14	42.57 ± 5.00	<0.001
BET (S)	50.10 ± 5.13	43.99 ± 5.54	0.005
BET (I)	51.35 ± 5.27	46.48 ± 8.83	0.014

CET: corneal epithelium thickness; LET: limbal epithelium thickness; BET: bulbar conjunctival epithelium thickness; N: nasal; T: temporal; S: superior; I: inferior.

**Table 3:** Statistical results of correlations between dry eye clinical tests.

Parameters	Gender	Age	OSDI	Schirmer 1 test	TBUT	Oxford scale	MCS
Age							
*r*	0.162						
*P*	0.136						
OSDI							
*r*	−0.048	0.071					
*P*	0.659	0.514					
Schirmer 1 test							
*r*	−0.098	−0.192	−0.312				
*P*	0.371	0.077	**0.003**				
TBUT							
*r*	−0.025	−0.201	−0.720	0.436			
*P*	0.817	0.063	**<0.001**	**<0.001**			
Oxford scale							
*r*	0.105	0.098	0.340	−0.274	−0.504		
*P*	0.337	0.370	**0.001**	**0.011**	**<0.001**		
MCS							
*r*	−0.155	−0.177	−0.137	0.111	0.211	−0.185	
*P*	0.155	0.103	0.209	0.309	0.054	0.089	
TFL							
*r*	0.044	0.053	0.163	−0.207	−0.236	−0.389	0.259
*P*	0.689	0.630	0.134	0.056	**0.029**	**<0.001**	**0.016**

DED: dry eye disease; OSDI: Ocular Surface Disease Index; TBUT: tear film break-up time; MCS: mean corneal sensitivity; TFL: tear film lipid layer interferometry.

**Table 4:** Correlations of ocular surface epithelial thickness with the result of clinical tests in the DED group.

Parameters	Gender	Age	OSDI	Schirmer I test	TBUT	Oxford scale	MCS	TFL
CET								
*r*	0.079	0.036	0.047	−0.109	−0.087	−0.104	−0.034	0.003
*P*	0.468	0.743	0.668	0.318	0.424	0.342	0.755	0.975
LET								
*r*	−0.178	−0.105	−0.305	0.263	0.378	−0.154	0.113	−0.081
*P*	0.252	0.504	**0.047**	0.089	**0.012**	0.325	0.469	0.607
LET (nasal)								
*r*	−0.043	−0.137	−0.322	0.209	0.311	−0.114	0.112	0.000
*P*	0.694	0.182	**0.022**	0.054	**0.004**	0.295	0.304	0.999
LET (temporal)								
*r*	−0.150	0.162	−0.169	0.059	0.280	0.062	0.003	−0.004
*P*	0.167	0.135	0.119	0.592	**0.009**	0.573	0.976	0.968
LET (superior)								
*r*	−0.001	−0.103	0.009	0.198	0.101	0.105	0.054	−0.043
*P*	0.995	0.343	0.933	0.067	0.354	0.335	0.623	0.694
LET (inferior)								
*r*	0.054	0.006	−0.519	0.271	0.638	−0.256	0.273	−0.066
*P*	0.429	0.953	**<0.001**	**0.012**	**<0.001**	**0.017**	**0.011**	0.548
BET								
*r*	0.157	0.085	0.362	−0.165	−0.428	0.134	−0.178	−0.105
*P*	0.314	0.588	**<0.001**	0.290	**<0.001**	0.392	0.252	0.504
BET (nasal)								
*r*	0.041	0.053	0.452	−0.116	−0.500	0.130	−0.105	−0.026
*P*	0.705	0.627	**<0.001**	0.254	**<0.001**	0.233	0.334	0.815
BET (temporal)								
*r*	0.023	0.295	0.497	−0.153	−0.479	0.132	−0.152	0.069
*P*	0.833	**0.006**	**<0.001**	0.326	**<0.001**	0.225	0.319	0.529
BET (superior)								
*r*	0.097	0.060	0.384	−0.075	−0.410	0.118	−0.286	−0.010
*P*	0.372	0.581	**<0.001**	0.493	**<0.001**	0.280	0.063	0.930
BET (inferior)								
*r*	0.191	−0.027	0.281	−0.049	−0.288	0.092	−0.184	0.097
*P*	0.078	0.807	**0.009**	0.651	**0.007**	0.401	0.089	0.376

DED: dry eye disease; OSDI: Ocular Surface Disease Index; TBUT: tear film break-up time; MCS: mean corneal sensitivity; TFL: tear film lipid layer interferometry; CET: corneal epithelium thickness; LET: limbal epithelium thickness; BET: bulbar conjunctival epithelium thickness.
